# Supplementary material for: Content-rich biological network constructed by mining PubMed abstracts
Source: BMC Bioinformatics. 2004 Oct 8;5:147. doi: 10.1186/1471-2105-5-147 (PMC528731; doi:10.1186/1471-2105-5-147)
Supplement: Additional File 2 — The original results of the above study (non-essential files are deleted to keep the file size under the limit set by BMC bioinformatics). [file 1471-2105-5-147-S2.bz2 › chilibotAdditionalFile2/dip30/760ID10756017E18146/html/DNAB_B.html]

 


 **DNAB** and **B** 
  
Found 69 abstracts in PubMed, retrieved 30.  
 

 What does Google say? 
 PDF only 
| .edu only 

---

**Interactive relationship** (e.g. stimulation, inhibition, etc)

**Neutral relationship**- In this paper we provide evidence for a functional interaction between the primosomal protein DnaI from  **B** .subtilis and the main hexameric replicative helicase < **b** > DnaB  **b** > from Bacillus stearothermophilus.  Ref: 11842108 Nucleic Acids Res, 2002
- Here we demonstrate that P2  **B**  associates with < **b** > DnaB  **b** >.  Ref: 10756017 J Virol, 2000
- DnaD alone and the DnaD < **b** > DnaB  **b** > pair interact specifically with PriA of  **B** . subtilis on several DNA substrates.  Ref: 11585815 J Biol Chem, 2001
- The interaction of bacteriophage P2  **B**  protein with Escherichia coli < **b** > DnaB  **b** > helicase.  Ref: 10756017 J Virol, 2000
- Even though P2  **B**  has little similarity to other known or predicted helicase loaders, we suggest that P2  **B**  is required for efficient loading of < **b** > DnaB  **b** >.  Ref: 10756017 J Virol, 2000
- These observations confirm that < **b** > DnaB  **b** >, DnaD and DnaI are primosomal proteins in  **B** . subtilis.  Ref: 11679082 Mol Microbiol, 2001
- A critical gene complex in  **B** . subtillis for the role of membrane complex is the < **b** > dnaB  **b** > operon that most likely consists of four genes < **b** > dnaB  **b** >, dnaI, ORFZ ORF213, and ORF omega ORF281.  Ref: 9427839 Prog Nucleic Acid Res Mol Biol, 1998
- This was done by using the yeast two hybrid system in vivo and was confirmed in vitro, where 35 S labeled P2  **B**  bound specifically to < **b** > DnaB  **b** > adsorbed to Q Sepharose beads and monoclonal antibodies directed against the His tagged P2  **B**  protein were shown to coprecipitate the < **b** > DnaB  **b** > protein.  Ref: 10756017 J Virol, 2000
- Other selected mutations that map to regions carrying Walker A and  **B**  boxes are expected to be defective in ATP binding, a required step in < **b** > DnaB  **b** > DnaC complex formation.  Ref: 11333269 J Biol Chem, 2001
- The role of the membrane on the termination and segregation of the daughter chromosomes has not been discovered, but an important clue comes from the terminus area of the  **B** . subtillis chromosome being bound to the membrane in a high salt resistant and < **b** > DnaB  **b** > independent fashion.  Ref: 9427839 Prog Nucleic Acid Res Mol Biol, 1998
- Here, we describe < **b** > dnaB  **b** > mutations that suppress the phenotypes of  **B** . subtilis priA mutants.  Ref: 11679082 Mol Microbiol, 2001
- Finally, P2  **B**  was shown to stabilize the opening of a reporter origin, a reaction that is facilitated by the inactivation of < **b** > DnaB  **b** >.  Ref: 10756017 J Virol, 2000

**Non-interactive relationship** (e.g. studied together, co-existance, homology, etc.)

- We have proposed previously that three proteins involved in the initiation of replication at oriC in  **B** . subtilis, < **b** > DnaB  **b** >, DnaD and DnaI, are components of the PriA primosome in this bacterium.  Ref: 11679082 Mol Microbiol, 2001
- Involvement of the  **B** .subtilis proteins < **b** > DnaB  **b** >, DnaD and DnaI, which do not have any known analogue in Escherichia coli, raises the possibility that primosome assembly and or function in  **B** .subtilis differs from that in E.coli.  Ref: 7781616 EMBO J, 1995
- The introduction into the plasmid of a hairpin structure, whose stem carries a DnaA box, mediates conversion of SS c into dsDNA and makes plasmid replication independent of the  **B** . subtilis < **b** > dnaB  **b** > function.  Ref: 8039651 FEMS Microbiol Lett, 1994
- ssiA priming activity requires the DnaE primase, the DnaC replication fork helicase, as well as the products of the < **b** > dnaB  **b** >, dnaD and dnaI genes of  **B** .subtilis, but not the RNA polymerase.  Ref: 7781616 EMBO J, 1995
- Our results indicate that the  **B** . subtilis dnaC gene encodes a counterpart of the E. coli < **b** > dnaB  **b** > helicase.  Ref: 7711902 Microbiology, 1995
- The sedimentation coefficient of the < **b** > DnaB  **b** > monomer s0 20, w = 2.8 0.3 S provides an apparent frictional ratio of 1.22 0.05, which indicates that the monomer has an elongated structure with an axial ratio of a  **b**  = 5.2 0.8 when modeled as a prolate ellipsoid of revolution.  Ref: 7989299 J Biol Chem, 1994
